# Endoparasite Infection Has Both Short- and Long-Term Negative Effects on Reproductive Success of Female House Sparrows, as Revealed by Faecal Parasitic Egg Counts

**DOI:** 10.1371/journal.pone.0125773

**Published:** 2015-05-01

**Authors:** Håkon Holand, Henrik Jensen, Jarle Tufto, Henrik Pärn, Bernt-Erik Sæther, Thor Harald Ringsby

**Affiliations:** 1 Centre for Biodiversity Dynamics, Department of Biology, Norwegian University of Science and Technology, NO-7491, Trondheim, Norway; 2 Centre for Biodiversity Dynamics, Department of Mathematics, Norwegian University of Science and Technology, NO-7491, Trondheim, Norway; Tuscia University, ITALY

## Abstract

Parasites have the potential to severely reduce host reproductive success. However, the effects of endoparasites on reproductive success have not received the same amount of attention as the effects of parasites on host survival. We investigated the relationship between an avian endoparasite (gapeworm, *Syngamus trachea*) and both current and future reproductive success of female house sparrows (*Passer domesticus*) in a population on the coast of Helgeland, northern Norway. We found that the proportion of eggs in a nest that failed to develop into fledglings increased as the faecal parasitic egg count of the mothers increased. We also found that juvenile females with high numbers of parasitic eggs in their faeces had lower lifetime reproductive success as adults. However, we did not find a relationship between maternal parasite infection and clutch size or recruitment rate of offspring. To our knowledge this is the first study to find a relationship between reproductive success of an avian host and faecal egg count of an endoparasite. The present study indicates that infection by an endoparasite may be associated with lower individual reproductive success in both the short-term and long-term in a wild population of hosts.

## Introduction

Parasites and diseases have the potential to reduce host fitness by their consumption of host resources. The effect of endoparasites on host fitness has often focused on survival or lifespan of the host and relatively less attention has been given to the influence on host reproduction (see [1–3]). If the strategy of the parasite does not utilize the reproductive stage of a host to its own benefit (e.g. transmission), the parasite may evolve mechanisms to limit host reproduction [3]. A parasite may achieve this by consuming energy that the host would otherwise have allocated to reproduction [1, 4, 5]. Parasite-induced reduction in host reproduction may be particularly favoured by parasites with a predominantly horizontal transmission (i.e. between individual hosts within a generation), allowing parasites to maximize the amount of energy consumed from the host without severely compromising host survival [3]. By minimizing the negative effect on host survival, parasites may also allow time and opportunity for the host to come into contact with potential new hosts before it dies.

The parasite may have an immediate effect on host reproduction in a given year or season (e.g. [6, 7]). Alternatively, there may be a cumulative effect of parasites on the host’s lifetime reproductive success (e.g. [8, 9]). However, the host may mount defences or allocate energy differently in response to parasite infection to compensate and minimize the cost of the infection in terms of host fitness [2, 10, 11]. For example, the host may delay current reproduction in favour of longer lifespan and a chance to compensate later in life [2, 11]. Thus the “arms race” between the host and the parasite may lead to a complex set of mechanisms with different strategies or trade-offs depending on the life history traits of the species. Theoretical population models also indicate that if a parasite affects host reproduction more than host survival, this may have destabilizing effects on the host-parasite dynamics and may increase the probability of extinction [12].

Life history theory suggests that negative effects (due to biotic and/or abiotic factors) suffered by individuals early in life may cause reduced reproductive success later in life [13]. For example, female red deer (*Cervus elaphus*) on the Isle of Rum, Scotland, born in years with high population densities start reproduction at a later age compared to females born in years with low population densities [14]. However, few studies have investigated if parasite infection may have similar long term negative effects on reproductive success in wild populations (but see [8, 9]).

In addition to possible effects of parasites on host reproduction, there may be several other important factors which affect individual host reproductive success (e.g. age or morphology), that should be accounted for in studies of potential effects of parasites on host fitness. For example, the reproductive success of individuals may decrease as they age (i.e. senescence) [15] or larger individuals may produce larger clutches [16]. Controlling for such effects requires detailed information on each individual included in the study. In addition, obtaining precise estimates of individual reproductive success in wild populations may be challenging. However, one study system which has managed to obtain estimates of several individual reproductive fitness components (clutch size, fledging failure and total number of recruits produced in a lifetime) is a metapopulation of house sparrows (*Passer domesticus*) on the coast of Helgeland in northern Norway [17–19]. In this system, data collected from both parents and offspring have made it possible to obtain different estimates of individual reproductive success along with individual covariates (age, morphology etc.). Our aim for this study was to investigate the relationship between individual maternal infection of the parasite *Syngamus trachea* in a given year and reproductive success. We also investigated the relationship between parasite infection (measured in faeces samples) when females were juveniles and their subsequent lifetime reproductive success.

## Material and Methods

### Study area

The study was carried out on the island of Hestmannøy (N 66° 33 00 E 12° 51 00), located in the Helgeland archipelago northern Norway (described in previous studies [20, 21]). Since 1993, house sparrows on Hestmannøy and the surrounding islands have been systematically captured, marked and reencountered several times during their lifetime (e.g. [19, 22, 23]). On Hestmannøy a high proportion of the individuals have been individually marked (> 90%). Faecal samples from house sparrows used for parasite egg counts have been collected on Hestmannøy since 2007 (see below).

### Study species

As described in detail previously [20, 21], the nematode worm *S*. *trachea* has a global distribution and has been found in a majority of terrestrial bird genera [24, 25]. The parasite is commonly known as “gapeworm” or “gapes” in the domestic bird industry, where previous outbreaks have been known to cause large problems [24]. The lifecycle of *S*. *trachea* may include one transport/paratenic host (i.e. a host which is not necessary for development of the parasite egg or larva) in addition to the final avian host. These transport/paratenic hosts can be species of earthworms, insects or and snails [24, 26]. The development-time from embryo to infective larvae is temperature dependant, with a lower limit of 16° C and an upper limit of 35° C [27]. As the body temperature of house sparrows are often above 35° C [28], this entails that eggs must develop outside the host in order to achieve transmission. Accordingly, vertical transmission (i.e. from parent to offspring) of *S*. *trachea* does not appear to be the main life history strategy of the parasite. After an avian host has orally ingested mature parasite larva(e), the parasite makes its way from the digestive system of the bird, through the blood vascular system to the lungs or trachea. Having reached the lungs or trachea, the parasite grows into an adult and finds a mate with which it permanently copulates [24]. The eggs produced by the adult parasite(s) are coughed up by the host, swallowed and passed in the faeces [24]. *S*. *trachea* feed on blood in the tracheal tissue and may cause anaemia, inflammation and mechanical damage to the tissue and excess mucus production in the lungs and trachea. Birds that are infected by *S*. *trachea* may develop visible symptoms consisting of coughing, gasping and shaking of the head. Discomfort caused by these symptoms may reduce food uptake, and combined with the blood loss, may lead to host mortality [24, 29]. Egg production of *S*. *trachea* in domestic species has been shown to peak in the first 40 days of infection [30]. The average life span of adult *S*. *trachea* in domestic bird species has been found to be 2–4 months [30]. A previous study of *S*. *trachea* in the study population also indicated that house sparrows very rarely become infected by *S*. *trachea* more than once in their lifetime [20].

The house sparrow is a small passerine bird with a global distribution and is common in the Northern hemisphere [28]. House sparrows live in close vicinity to human settlements [28]. House sparrows on Hestmannøy live in the vicinity of dairy farms and build their nests inside barns, silos or underneath ceilings. The survival probability of house sparrows is generally found to be low. This means that even if a house sparrow survives to become an adult, few survive more than two years [28].

### Ethical statement

The research was carried out in accordance with permits (Permit Number: 448.48/0) from the Bird Ringing Centre at Stavanger Museum, Norway, on behalf of the Norwegian Environment Agency and in accordance with Norwegian legal policy for animal welfare approved by the Norwegian Animal Research Authority (Permit Number: FOTS 6475). These permits cover all research activity involving animals described in this study. No animals were killed/sacrificed in the course of this study.

### Field work, sampling and analysis

Adult and juvenile house sparrows were captured using mist nets. Upon first capture, they were banded with a metal ring engraved with a unique id-number and three plastic colour rings (two rings on each tarsus). Tarsus length (to nearest 0.01 mm), body mass (to nearest 0.1 g), bill (length and depth, to nearest 0.01 mm) and wing length (to nearest mm) were measured. A body condition index (BC) was estimated as the unstandardized residuals obtained by regressing body mass on tarsus length with a linear model (all years pooled), i.e. individual body mass accounting for the skeletal body size. Variation in measurements among fieldworkers was accounted for by obtaining a relationship between each individual fieldworker and the most experienced fieldworker by using general linear regression techniques (see [20, 21, 31, 32] for detailed description of field work). In addition we visited nests at least once per week and counted the number of eggs and chicks and their age. When a house sparrow had been captured in a mist net, it was placed in a paper bag with a flat bottom and with a small hole at the top to prevent asphyxiation. The bird was placed in the paper bag as soon as possible after capture (1–5 minutes). A faecal sample was obtained by the bird defecating in the paper bag. The sample was then placed in a 1.5 mL cryo-tube filled with 1 mL of MilliQ H_2_O. The samples were stored at temperatures of 1–8°C until they could be analysed (max. 12 months after collection). Eggs of *S*. *trachea* stored for 12 months were found to be viable (i.e. L3-larvae could be cultivated successfully).

During the analysis of an individual faecal sample, the sample was first centrifuged at 605 g for 60 seconds in an Eppendorf Minispin [20]. The MilliQ H_2_O was then removed and replaced with sucrose-saturated MilliQ H_2_O (sp. Gravity = 1.28) [20]. Due to the small volume of individual faecal samples and hence small variation in mass between them, all faecal samples were analysed in the same volume of sucrose solution. The sample was then centrifuged again at 151 g for 45 seconds [20]. The entire content of the cryotube was then placed in a Mcmaster/whitlock counting chamber under a microscope (Leica, model DMLS) [20].The total number of *S*. *trachea* eggs contained in the sample was then counted (faecal egg count, FEC). The eggs of *S*. *trachea* were identified by their characteristic shape (bipolar and visible opercula at both ends) and size (approximately 80–100 μm × 50–60 μm) [21, 24]. The visible opercula and length of the eggs made it possible to identify eggs that were distorted or damaged by the flotation liquid or other causes. The presence of *S*. *trachea* in the population of house sparrows has been confirmed by post-mortem examinations by the Norwegian Veterinary Institute (adult parasite- and egg morphology) of birds (*n* = 2) that died during handling (prior to this study) due to blockage of the trachea by the parasite (Unpublished results). The repeatability (0.97) of the faecal egg counting procedure has been found to be very high [21]. A previous study also found that house sparrows exhibiting symptoms of infection (i.e. gasping for air) had a significantly higher mean FEC compared to non-symptomatic individuals [21].

Our first dataset was used for investigations of reproductive success of adult female house sparrows in a given clutch and consisted of observations of 257 eggs and 153 fledglings in 53 nests where all 53 mothers (46 unique birds) were sampled for faeces during the summer of 2007–2011 (01 May—15 August). Our second dataset was used to investigate individual lifetime reproductive success of female house sparrows and contained 55 faeces samples from 42 juvenile females collected in the summer and autumn of 2007–2009 (01 May—07 October). Estimation of reproductive success was based on genetic parentage analyses of DNA from blood samples (see S1 Appendix, for detailed description of methods).

### Statistical analyses

All statistical analyses were conducted in the statistical software R version 2.15.3 [33].

#### Maternal infection and current breeding attempt

We first investigated the relationship between maternal infection and reproductive success measured as either clutch size (number of eggs), fledging failure or recruitment failure (first dataset, see above). To test if there was a mean difference between infected/non-infected mothers or a linear relationship with faecal egg count (FEC), we used maternal infection status (no/yes) or FEC as explanatory variables. We included females which had been captured and sampled during the period when their nest contained eggs or chicks. This period corresponds to hatch date ± 14 days, as egg laying and incubation may take 14 days [28] and chicks generally leave the nest when they are 14 days old [28]. In addition to infection status and FEC, we included clutch number, clutch size, hatch day, hatch day^2^, calendar year (as a factor) and the mothers; age, age^2^ and morphology (BC, bill depth, bill length, tarsus length and wing length) as explanatory variables. Because 6 birds appeared as mothers more than once in our dataset, we used maternal identity as a random factor in all models in order to avoid pseudoreplication.

The first analysis focused on the relationship between clutch size (measured as the number of eggs laid by a female) and infection. In this analysis we used the clutch size as the response variable in generalized linear mixed models (package lme4, function = glmer, family = poisson, link = log). In the second part of our first section, we investigated the probability of fledging failure measured as the proportion of eggs in a nest that failed to develop into fledglings (nestlings with age 9–13 days) by using generalized linear mixed models (package lme4, function = glmer, family = binomial, link = cloglog). In these models, we also included the age of fledglings (9–13) as an offset in all models to account for differences in survival intervals from egg to fledgling [34]. We also weighted the proportion of fledgling failure in a given nest by the number of eggs present in the nest (i.e. number of trials). Lastly in our first section, we investigated the probability of fledglings failing to become recruits (i.e. probability of fledglings in a given nest that failed to survive their first winter) by using generalized linear mixed models (package lme4, function = glmer, family = binomial, link = cloglog). We only included nests that produced fledglings (n = 51). We also weighted the proportion of recruitment failure in a given nest by the number of fledglings present in the nest (i.e. number of trials). We thus investigated if maternal infection could have a delayed relationship with mortality of offspring after fledging.

#### Lifetime reproductive success and juvenile infection

Our second section of analyses investigated the relationship between female lifetime reproductive success (LRS) and their infection status or FEC as a juvenile. In these analyses we used our second dataset (see above) which was collected from captured female juveniles and only included female juveniles that survived to become adults. None of these individuals were observed or captured in 2013 and were assumed to be dead after 2012. Previous analyses [21] have found a very high mean resighting probability (~ 0.9) on Hestmannøy. Accordingly, we assumed that the estimated total number of recruits produced per female 2007–2012 represented an estimate of the LRS for these individuals. For these analyses we used generalized linear models (function = glm, family = poisson, link = log) with LRS as the response variable. As explanatory variables we used FEC, infection status and juvenile morphology (BC, bill depth, bill length, tarsus length and wing length). In addition we included the lifespan (number of years) and the hatch year of individuals as explanatory variables.

All candidate models were ranked using Akaike information criterion corrected for small sample sizes (AIC_C_), which penalizes models with a high number of parameters relative to sample size [35]. We also calculated the Akaike weights [35] of all candidate models. Estimates from candidate models are reported as mean ± 1 standard error. In order to prevent any problems due to multicollinearity, all explanatory variables were assessed for collinearity visually, by the use of Pearson’s correlation coefficient (r_p_) and variance inflation factors (VIF). Models with higher-order terms also included the respective main effects (e.g. age and age^2^). To avoid overparametrization [35], we did not include models with a sample size to variable ratio < 10 (i.e. never less than 10 samples per variable). Due to the relatively small size of our datasets, we did not include interactions between explanatory variables in any models. We also did not allow FEC and infection status to feature together in the same candidate model(s) due to their collinearity.

## Results

### Maternal infection and current breeding attempt

In the analysis of variation in clutch size, the highest ranked model contained only the intercept; i.e. none of our explanatory variables. There were also four alternative models which had a ∆ AIC_C_ < 2 (S1 Table). These four models were univariate and only contained the effect of either bill length, wing length, age or body condition, respectively.

**Fig 1:**
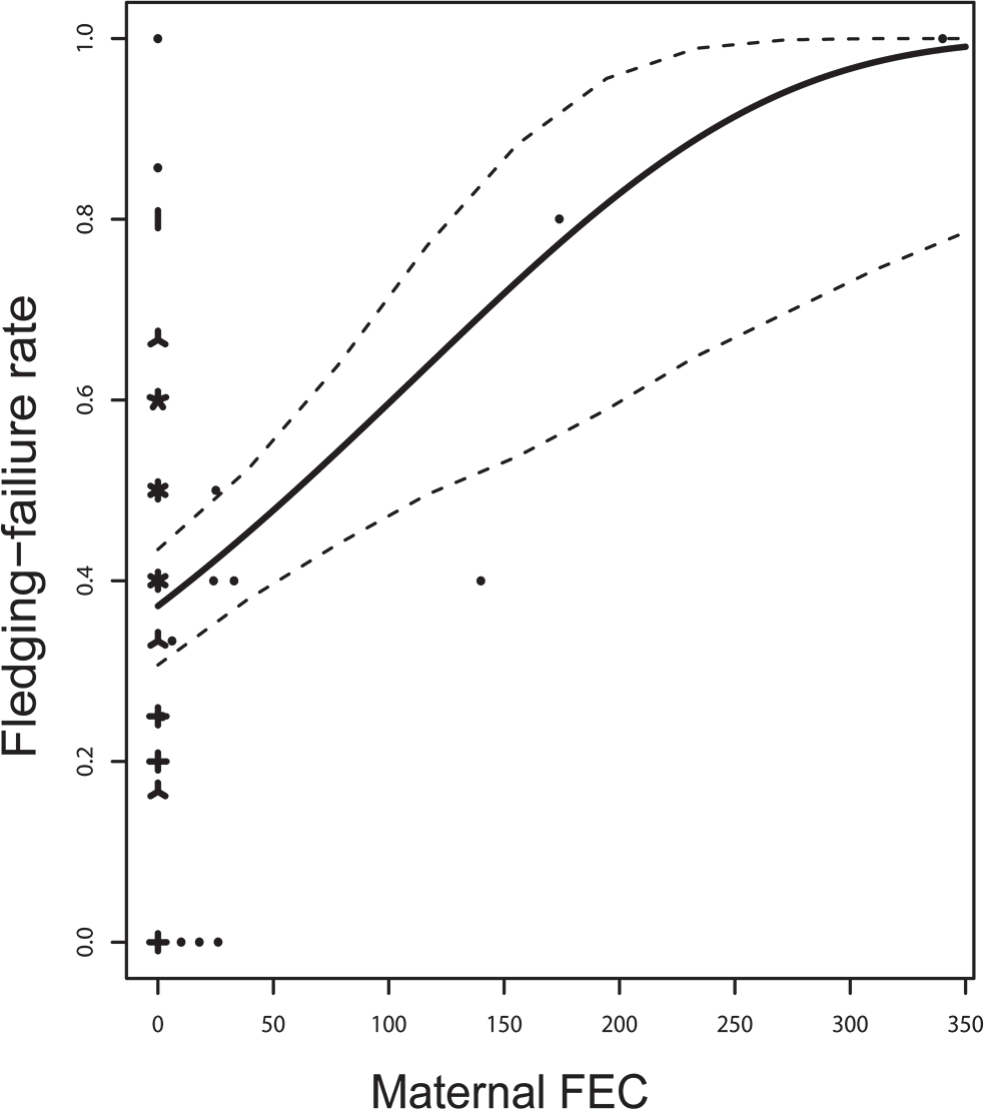
Fledgling failure and maternal infection. The relationship between fledgling failure (i.e. probability that eggs fail to develop into fledglings) over a 11-day interval with maternal faecal egg count (FEC) of the parasite *Syngamus trachea* in house sparrows on the island of Hestmannøy, northern Norway. The solid line indicates predicted values from the highest ranked generalized linear mixed model as judged by AIC_C_ (see S2 Table). The model included individual identity as a random factor, and maternal tarsus length and hatch day as fixed covariates. Dotted lines indicate lower and upper limits of the 95% confidence interval of predicted values. “Sunflower” points indicate sample size of observed values.

Fledging failure was best explained (i.e. lowest AIC_C_) by FEC of the mother, hatch day and tarsus length of the mother. Fledging failure was higher for mothers with higher FECs (0.007 ± 0.002, see Fig 1), mothers breeding later in the breeding season (- 0.017 ± 0.005) and for mothers with shorter tarsus length (- 0.286 ± 0.092). The seven models with ∆ AIC_C_ < 2 all contained a positive relationship between fledging failure and FEC, in addition to the additive effects of hatch day and tarsus length (S2 Table). The highest ranked model which included infection status (rank = 148, ∆ AIC_C_ = 11.51), contained an uncertain estimate of the relationship between fledging failure and maternal infection status (0.235 ± 0.311).

Recruitment failure was best explained by a model that contained only the intercept; i.e. none of the explanatory variables were included. However there were eight other candidate models which did not obtain a substantially higher AIC_C_ value (∆ AIC_C_ < 2) compared to the highest ranked model (S3 Table). Although one of these models contained FEC of the mother, the age and wing length of the mother, and hatch day appeared to be of higher importance.

### Lifetime reproductive success and juvenile infection

**Fig 2:**
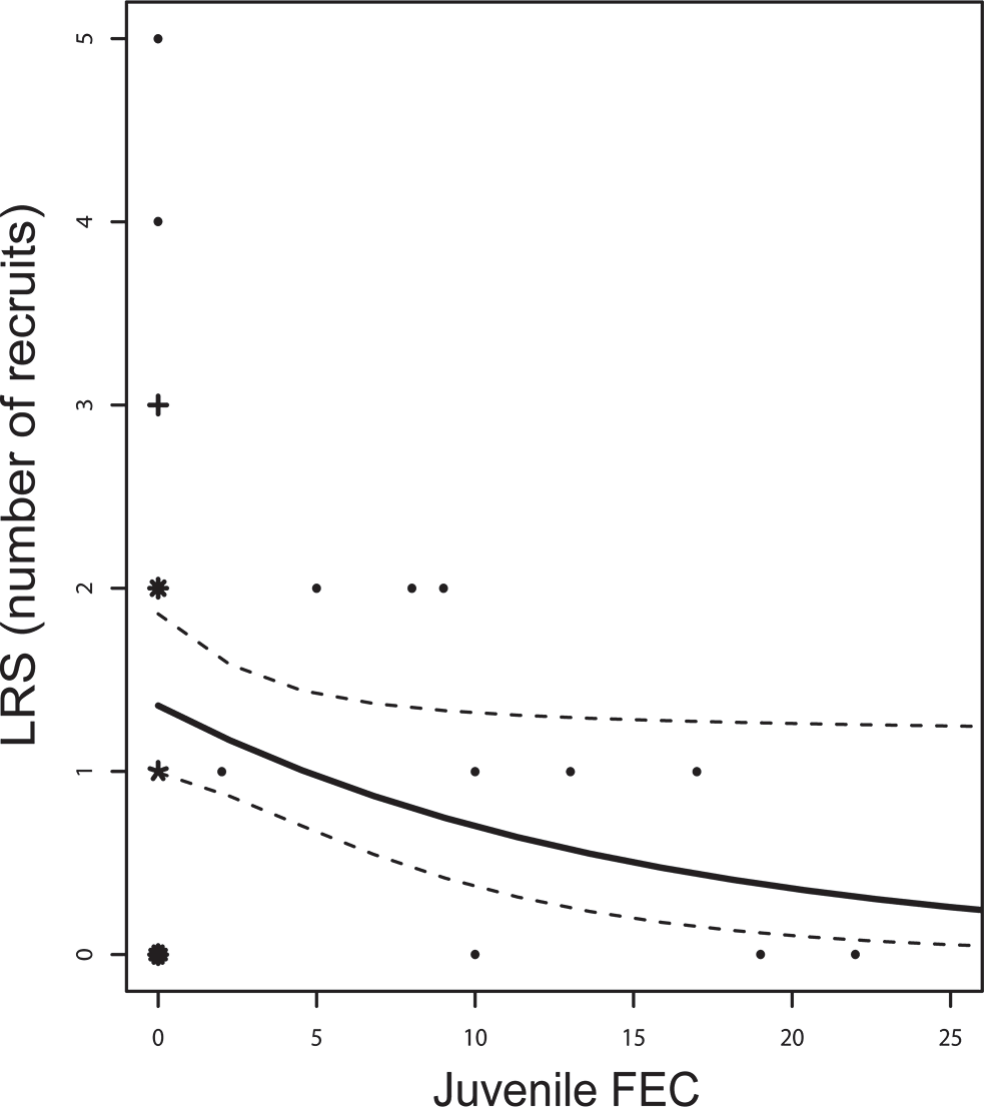
Lifetime reproductive success and juvenile infection. The predicted relationship between faecal egg count (FEC) of the parasite *Syngamus trachea* and individual lifetime reproductive success of juvenile female house sparrows on the island of Hestmannøy, northern Norway, measured as the number of recruits produced in an individual’s lifetime. The solid line indicates predicted values from the highest ranked generalized linear mixed model as judged by AIC_C_ (see S4 Table). Dotted lines indicate lower and upper limits of the 95% confidence interval of predicted values. “Sunflower” points indicate sample size of observed values (one observation omitted to improve clarity).

LRS was best explained by a model that included the lifespan, juvenile FEC and bill depth of the individual female. LRS was highest for individuals with a long lifespan (0.416 ± 0.146), large juvenile bill depth (1.249 ± 0.567) and low juvenile FEC (- 0.066 ± 0.033, see Fig 2 and S4 Table). All of the seven models with ∆ AIC_C_ < 2 contained the effect of lifespan and FEC. The highest ranked model which included infection status (rank = 14, ∆ AIC_C_ = 2.57) contained a negative estimate of the relationship between juvenile infection and LRS (- 0.634 ± 0.357).

## Discussion

This study has found a negative relationship between reproductive success of female house sparrows and the number of parasite eggs found in faeces in an insular population on the coast of Helgeland in northern Norway. A negative relationship with FEC was found both for reproductive success of adult females in a given year (measured as fledging failure) and adult reproductive success of juvenile females (LRS). To our knowledge this is the first study to find a negative relationship between FEC of a parasite eggs and reproductive success in wild populations of avian hosts. However, Redpath et al. [36] showed that experimental reductions of parasite infections led to an increased brood size in red grouse (*Lagopus lagopus scoticus*). Correlations between faecal larval counts and reproductive success of the host have also been found in red deer [37]. Combining the results of the current study with the results of Holand et al. [21] suggest that parasite infection may increase variation in individual fitness in the metapopulation. However, to fully investigate the role of parasites in population dynamics of hosts, experimental manipulation of parasite prevalence has been suggested as vital in order to demonstrate the effect of parasites [38, 39].

The relationship between faecal egg counts and infection intensity may not be straightforward. As faecal egg counts may vary over the lifespan of the parasite [30], this may have introduced variation in the relationship between the number of adult parasites present in the host and FEC. Ideally, the number of adult parasites present in a given host should be estimated by post mortem examinations. This was not possible for this long term, non-invasive study. In addition, we were unable to standardize the egg count (i.e. egg per gram) due to the generally very low mass of faeces samples produced by house sparrows. This may have caused unexplained variation in the relationship between FEC and response variables. However, repeated samples (20 samples over 40 days) from an infected house sparrow under laboratory conditions (Holand et al. unpublished results) indicated a strong positive relationship between standardized egg counts and FEC (*r*_*p*_ = 0.85, *df* = 18, *P* < 0.0001). In addition, the positive relationship between symptoms of infection and FEC [21] may be explained by a correlation between FEC and infection severity/intensity.

The apparent lack of support in our models for the importance of infection status (compared to FEC) may be expected if infected hosts vary substantially in their parasite load [40]. Therefore, the effect of classifying hosts only as “infected” or “not infected” may not optimally reflect the variation within the “infected” group. Given that FEC reflects the variation in the “infected” group better than the simple categorical variable, this may explain why FEC was generally favoured more than infection status in the model selection.

We did not find evidence for a relationship between infection by *S*. *trachea* and clutch size. Indeed, none of our explanatory variables were present in the highest ranked model (S1 Table). Clutch size in house sparrows has been found to vary with time of year, habitat, density and female age [28]. However, the general strength of these relationships appears to vary among studies [28]. A lack of a relationship between infection by *S*. *trachea* and clutch size may be expected as egg production and egg laying in passerines may be energetically less costly than post-hatch parental care [41]. If the parasite reduces the amount of energy available to the host for reproduction, this effect may not be as strong in the less costly stages (i.e. egg production) of host reproduction (but see [42]).

The variation in fledging failure of eggs in nests was most parsimoniously explained by the additive effects of maternal FEC, hatch day and maternal tarsus length (S2 Table). That fledging failure increased as the maternal FEC increased (Fig 1) suggests that nestlings suffered higher mortality if the mother was heavily infected. This could be caused by a reduction in energy available to the mother in caring for the offspring. Female house sparrows in our study system have been shown to have higher feeding rate of nestlings compared to males [32]. Therefore, if the female suffered significantly reduced energy reserves due to parasite infection, this could potentially increase nestling mortality. Also, in a study of survival rates using capture-mark-recapture models we found that individuals with symptoms of infection by *S*. *trachea* had lower reencounter probability, compared to non-infected house sparrows [21]. If severe infection by *S*. *trachea* caused a reduction in the level of host activity, this could explain both the increased fledging failure and lower reencounter probability. Because our analysis contained observations of 46 individuals in total, where 11 mothers were infected, one should be cautious in interpreting the generality of the results (see Fig 1). Nevertheless, we did not find any reason to doubt the validity of these observations (e.g. due to measurement errors or other special circumstances). Few experimental studies have demonstrated an increased fledging failure in birds due to maternal infection by endoparasites. However, Marzal et al. [6] found that adult house martins (*Delichon urbica*) treated for avian malaria (*Haemoproteus prognei*) produced more fledglings compared to untreated adults. Also, negative effects of parental infection on offspring survival have been found in the European shag (*Phalacrocorax aristotelis*) infected by endoparasites [43].

We found no clear relationship between recruitment failure and maternal characteristics (including FEC and infection status) or brood characteristics such as hatch day. This suggests that after a fledgling has left the nest, its survival may depend more on other factors which are not conditional on the nest or mother. For example, a previous investigation in the study system [21] found that survival probability of juveniles and adults decreased as population density increased.

We also found that LRS of juvenile females decreased with increasing juvenile FEC after accounting for the effect of lifespan and tarsus length (Fig 2). This suggests that infection of *S*. *trachea* when the individual was juvenile had a negative effect on reproductive success as an adult. Negative effects of parasite infection on LRS have previously been found in cliff swallows (*Hirundo pyrrhonota*) infected by ectoparasites [8]. In addition, Fitze et al. [9] found that Great tit (*Parus major*) nestlings that grew up in nests containing ectoparasites had a lower lifetime production of reproducing offspring compared to nestlings that grew up in uninfected nests. However, to our knowledge the current study is the first to find a relationship between adult reproductive success and juvenile FEC of an avian host. As only 15 of the juveniles had a known age (i.e. had been marked as fledglings), we were unable to account for the effects of development on measurements of juvenile morphology. This may have added variation in the relationships between LRS and juvenile morphology.

This study has found negative relationships between parasite infection, measured as eggs in faeces, and host reproduction that could indicate a negative effect of parasites on host reproductive success in a given year and in reproductive success as an adult (LRS) of a host individual. Whether the pattern found in this study was caused by an adaptive exploitation of the host by the parasite, a trade off strategy by the host or simply a consequence of a general loss of energy/resources caused by the infection, should be a topic for further investigations, ideally using an experimental design. Both the underlying (co)evolutionary mechanisms and ecological impact of parasite-induced reduction in host fitness has become an area of increased interest in recent years due to the possible effect of climate change on these mechanisms [44]. Understanding the causes and consequences of such effects could have considerable impact for our understanding of the population dynamics of both parasite and host species.

## Supporting Information

S1 Appendix Methods for estimation of reproductive success. (DOCX)

S1 Table Highest ranked models (based on AIC_C_) of clutch size. Table of the highest ranked models in an AICC comparison of generalized linear mixed models explaining clutch size of adult female house sparrows on the coast of Helgeland in northern Norway. The table shows the parameter estimates ± 1 standard error of the explanative variables included in the models. All models included maternal identity as a random factor. Data was collected on the island of Hestmannøy during the years 2007–2011. Morphological variables and age are from the mother of the clutch. Variable importance is given for each variable in parenthesis below the variable name (based on models with ∆ AIC_C_ < 2).
(DOCX)

S2 Table Highest ranked models (based on AIC_C_) of fledging failure. Table of the highest ranked models in an AIC_C_ comparison of generalized linear mixed models of fledging failure in house sparrows on the coast of Helgeland in northern Norway. The table shows the parameter estimates ± 1 standard error of the explanative variables included in the models. All models included maternal identity as a random factor and the age of fledglings as an offset. Data was collected on the island of Hestmannøy during the years 2007–2011. Morphological variables and age are from the mother of the offspring. FEC was the number of eggs from the parasite *Syngamus trachea* that was found in the faeces of the mother during the period when the eggs/offspring was in the nest. Variable importance is given for each variable in parenthesis below the variable name (based on models with ∆ AIC_C_ < 2).
(DOCX)

S3 Table Highest ranked models (based on AIC_C_) of recruitment failure. Table of the highest ranked models in an AIC_C_ comparison of generalized linear mixed models of recruitment failure of fledgling house sparrows on the coast of Helgeland in northern Norway. The table shows the parameter estimates ± 1 standard error of the explanative variables included in the models. All models included maternal identity as a random factor. Data was collected on the island of Hestmannøy during the years 2007–2011. Morphological variables and age are from the mothers of the offspring. FEC was the number of eggs from the parasite *Syngamus trachea* that was found in the faeces of the mother during the period when the eggs/offspring were in the nest. Variable importance is given for each variable in parenthesis below the variable name (based on models with ∆ AIC_C_ < 2).
(DOCX)

S4 Table Highest ranked models (based on AIC_C_) of lifetime reproductive success. Table of the highest ranked models in an AIC_C_ comparison of generalized linear models of lifetime reproductive success of juvenile female house sparrows on the coast of Helgeland in northern Norway born in the years 2007–2009. The table shows the parameter estimates ± 1 standard error of the explanative variables included in the models. Data on reproductive success was collected on the island of Hestmannøy during the years 2007–2012. FEC was the number of eggs from the parasite *Syngamus trachea* that was found in the faeces of the juvenile females. “×” denotes the presence of a given factor in a given model. Variable importance is given for each variable in parenthesis below the variable name (based on models with ∆ AIC_C_ < 2).
(DOCX)
