# Supplementary material for: Endoparasite Infection Has Both Short- and Long-Term Negative Effects on Reproductive Success of Female House Sparrows, as Revealed by Faecal Parasitic Egg Counts
Source: PLoS One. 2015 May 1;10(5):e0125773. doi: 10.1371/journal.pone.0125773 (PMC4416917; doi:10.1371/journal.pone.0125773)
Supplement: S1 Appendix — (DOCX) [file pone.0125773.s001.docx]

**S1 Appendix. Methods for estimation of reproductive success.**

Genetic parentage was determined from microsatellite analyses of DNA from blood samples. We collected a small blood sample (25 µl) from captured birds (adults and juveniles) and from nestlings. The blood samples were preserved in tubes containing 1 ml of 96 % ethanol. A small amount of the collected blood was lysed in 60 µl Lairds buffer [[1](#_ENREF_1)], with 90 µg proteinase K (Sigma Aldrich, St Louis, MO, USA), and incubated at 50°C for 3 hours. Genomic DNA was extracted from the lysate using the ReliaPrep Large Volume HT gDNA Isolation System (Promega, Madison, WI, USA), automated on a Biomek NXp robot (Beckman Coulter, Miami, FL, USA) and following the manufacturer’s recommendations; the only exception being elution of DNA in 25 mM Tris HCl (pH 8). The DNA concentrations were measured using a Fluostar Omega scanner (MBG Labtech, Offenburg, Germany). Samples with concentrations above 60 ng/µl were normalized to a concentration of 50 ng/µl with 25 mM Tris HCl (pH 8). The DNA was used in genotyping of each individual on fourteen fluorescently labeled (forward primers) and highly polymorphic microsatellite loci by polymerase chain reaction (PCR) in two multiplex panels. Panel 1: Ase18 [[2](#_ENREF_2)], Pdoµ1, Pdoµ3 [[3](#_ENREF_3)], Pdoµ5, [[4](#_ENREF_4)], Pdo10, Pdo33, Pdo40 [[5](#_ENREF_5)]. Panel 2: Pdo16, Pdo17, Pdo19, Pdo22, Pdo27, Pdo44, Pdo47 [[5](#_ENREF_5)]. A “pigtail” (GTTT) was added to the 5’ end of all reverse primers. [[PCR, see 6 for further details](#_ENREF_6)]. PCR amplification of the microsatellite loci was carried out using the QIAGEN Multiplex PCR kit (QIAGEN GmbH, Germany) in 10 µl reactions containing 5 µl QIAGEN multiplex solution, 2 µl primer mix and 3 µl DNA solution. To visualize alleles, reverse primers were fluorescently labeled with FAM (Pdoμ1, Pdoμ5, Pdo19, Pdo22, and Pdo44; Invitrogen), NED (Pdoμ3, Pdo16, Pdo27 and Pdo33; Applied Biosystems), VIC (Ase18, Pdo10, Pdo40, and Pdo47; Applied Biosystems) or PET (Pdo17; Applied Biosystems). We ran touch-down PCR profiles on GeneAmp PCR system 9700 (Applied Biosystems, USA), starting with 94 °C for 15 minutes, 12 cycles with 94 °C for 30 seconds, 1 minute 30 seconds at the annealing temperature (one degree decrease each cycle, starting at 62 °C), and 72 °C for 1 minute. These touch-down steps were followed by 19 cycles with 94 °C for 30 seconds, 50 °C for 1 minute 30 seconds, and 72 °C for 1 minute. The final step was 5 minutes at 60 °C. PCR products (1 µl) were mixed with 10 µl HiDi formamide and 0.5 µl GeneScan LIZ600 size standard (Applied Biosystems, USA) and separated by electrophoresis in an automated 16 capillary electrophoretic analysis system: ABI Prism 3130xl Genetic Analyzer (Applied Biosystems). The resulting genotypes were scored using GeneMapper 4.0 (Applied Biosystems, USA). For genetic parentage analyses we used the software Cervus version 3.0 [[7](#_ENREF_7)]. One year was analyzed at the time to find genetic parents of all nestlings and fledged juveniles. All adult individuals recorded as present in the given year (i.e. recorded that year, or in an earlier *and* a later year) were defined as candidate parents. We used 90% as our confidence limit and assumed that the proportion of candidate parents sampled was approximately 90% to allow for immigrants and any unrecorded adults present in the population. Furthermore, the genotyping error rate was assumed to be 2.5% (based on observed error rates in duplicated samples from 570 individuals), and the level of relatedness (calculated based on the pedigree) among candidate parents was taken into account (see [[6](#_ENREF_6), [8](#_ENREF_8)] for further details on genotyping and parantage analyses).

**References**

1. Ausubel FM, Brent R, Kingston RE, Moore DD, Seidman JG, Struhl K. Current Protocols in Molecular Biology. New York: John Wiley & Sons; 1989.

2. Griffith SC, Dawson DA, Jensen H, Ockendon N, Greig C, Neumann K, et al. Fourteen polymorphic microsatellite loci characterized in the house sparrow *Passer domesticus* (Passeridae, Aves). Mol Ecol Notes. 2007;7(2):333-6. doi: 10.1111/j.1471-8286.2006.01598.x. PubMed PMID: WOS:000244520600043.

3. Neumann K, Wetton JH. Highly polymorphic microsatellites in the house sparrow *Passer domesticus*. Mol Ecol. 1996;5(2):307-9. PubMed PMID: WOS:A1996UM93000016.

4. Griffith SC, Stewart IRK, Dawson DA, Owens IPF, Burke T. Contrasting levels of extra-pair paternity in mainland and island populations of the house sparrow (*Passer domesticus*): is there an 'island effect'? Biol J Linnean Soc. 1999;68(1-2):303-16. doi: 10.1111/j.1095-8312.1999.tb01171.x. PubMed PMID: WOS:000083032600018.

5. Dawson DA, Horsburgh GJ, Krupa AP, Stewart IRK, Skjelseth S, Jensen H, et al. Microsatellite resources for Passeridae species: a predicted microsatellite map of the house sparrow *Passer domesticus*. Mol Ecol Resour. 2012;12(3):501-23. doi: 10.1111/j.1755-0998.2012.03115.x. PubMed PMID: WOS:000302552900017.

6. Jensen H, Sæther B-E, Ringsby TH, Tufto J, Griffith SC, Ellegren H. Sexual variation in heritability and genetic correlations of morphological traits in house sparrow (*Passer domesticus*). J Evol Biol. 2003;16(6):1296-307. doi: 10.1046/j.1420-9101.2003.00614.x. PubMed PMID: ISI:000185988000024.

7. Kalinowski ST, Taper ML, Marshall TC. Revising how the computer program CERVUS accommodates genotyping error increases success in paternity assignment. Mol Ecol. 2007;16(5):1099-106. doi: 10.1111/j.1365-294X.2007.03089.x. PubMed PMID: WOS:000244245300014.

8. Jensen H, Sæther B-E, Ringsby TH, Tufto J, Griffith SC, Ellegren H. Lifetime reproductive success in relation to morphology in the house sparrow *Passer domesticus*. J Anim Ecol. 2004;73(4):599-611. PubMed PMID: ISI:000222051300001.
