# Supplementary material for: Endoparasite Infection Has Both Short- and Long-Term Negative Effects on Reproductive Success of Female House Sparrows, as Revealed by Faecal Parasitic Egg Counts
Source: PLoS One. 2015 May 1;10(5):e0125773. doi: 10.1371/journal.pone.0125773 (PMC4416917; doi:10.1371/journal.pone.0125773)
Supplement: S3 Table — Table of the highest ranked models in an AICC comparison of generalized linear mixed models of recruitment failure of fledgling house sparrows on the coast of Helgeland in northern Norway. The table shows the parameter estimates ± 1 standard error of the explanative variables included in the models. All models included maternal identity as a random factor. Data was collected on the island of Hestmannøy during the years 2007–2011. Morphological variables and age are from the mothers of the offspring. FEC was the number of eggs from the parasite Syngamus trachea that was found in the faeces of the mother during the period when the eggs/offspring were in the nest. Variable importance is given for each variable in parenthesis below the variable name (based on models with ∆ AICC < 2). (DOCX) [file pone.0125773.s004.docx]

**S3 Table. Highest ranked models (based on AIC_C_) of recruitment failure.**

Table of the highest ranked models in an AIC_C_ comparison of generalized linear mixed models of recruitment failure of fledgling house sparrows on the coast of Helgeland in northern Norway. The table shows the parameter estimates ± 1 standard error of the explanative variables included in the models. All models included maternal identity as a random factor. Data was collected on the island of Hestmannøy during the years 2007-2011. Morphological variables and age are from the mothers of the offspring. FEC was the number of eggs from the parasite *Syngamus trachea* that was found in the faeces of the mother during the period when the eggs/offspring were in the nest. Variable importance is given for each variable in parenthesis below the variable name (based on models with ∆ AIC_C_ < 2).

| No. | Intercept | Age  (0.33) | Wing length  (0.23) | Hatch day  (0.17) | FEC  (0.09) | Bill length  (0.08) | Clutch size  (0.08) | Bill depth | ∆ AIC_C_ | Weight |
| --- | --- | --- | --- | --- | --- | --- | --- | --- | --- | --- |
| 1 | 0.61 ± 0.12 |  |  |  |  |  |  |  | 0 | 0.02 |
| 2 | 0.86 ± 0.22 | - 0.15 ± 0.11 |  |  |  |  |  |  | 0.49 | 0.02 |
| 3 | 0.63 ± 0.12 |  | - 0.09 ± 0.07 |  |  |  |  |  | 0.82 | 0.02 |
| 4 | 0.59 ± 0.12 |  |  | 0.005 ± 0.006 |  |  |  |  | 1.45 | 0.01 |
| 5 | 0.88 ± 0.22 | - 0.15 ± 0.11 | - 0.08 ± 0.07 |  |  |  |  |  | 1.47 | 0.01 |
| 6 | 0.59 ± 0.13 |  |  |  | 0.005 ± 0.006 |  |  |  | 1.58 | 0.01 |
| 7 | 0.61 ± 0.12 |  |  |  |  | 0.25 ± 0.36 |  |  | 1.83 | 0.01 |
| 8 | 1.00 ± 0.60 |  |  |  |  |  | - 0.08 ± 0.12 |  | 1.87 | 0.01 |
| 9 | 0.85 ± 0.22 | - 0.15 ± 0.11 |  | 0.005 ± 0.006 |  |  |  |  | 1.94 | 0.01 |
| 10 | 0.61 ± 0.12 |  |  |  |  |  |  | 0.31 ± 0.57 | 2.01 | 0.01 |
|  |  |  |  |  |  |  |  |  |  |  |
