# Supplementary material for: Endoparasite Infection Has Both Short- and Long-Term Negative Effects on Reproductive Success of Female House Sparrows, as Revealed by Faecal Parasitic Egg Counts
Source: PLoS One. 2015 May 1;10(5):e0125773. doi: 10.1371/journal.pone.0125773 (PMC4416917; doi:10.1371/journal.pone.0125773)
Supplement: S4 Table — Table of the highest ranked models in an AICC comparison of generalized linear models of lifetime reproductive success of juvenile female house sparrows on the coast of Helgeland in northern Norway born in the years 2007–2009. The table shows the parameter estimates ± 1 standard error of the explanative variables included in the models. Data on reproductive success was collected on the island of Hestmannøy during the years 2007–2012. FEC was the number of eggs from the parasite Syngamus trachea that was found in the faeces of the juvenile females. “×” denotes the presence of a given factor in a given model. Variable importance is given for each variable in parenthesis below the variable name (based on models with ∆ AICC < 2). (DOCX) [file pone.0125773.s005.docx]

**S4 Table. Highest ranked models (based on AIC_C_) of lifetime reproductive success.**

Table of the highest ranked models in an AIC_C_ comparison of generalized linear models of lifetime reproductive success of juvenile female house sparrows on the coast of Helgeland in northern Norway born in the years 2007 - 2009. The table shows the parameter estimates ± 1 standard error of the explanative variables included in the models. Data on reproductive success was collected on the island of Hestmannøy during the years 2007 - 2012. FEC was the number of eggs from the parasite *Syngamus trachea* that was found in the faeces of the juvenile females. “×” denotes the presence of a given factor in a given model. Variable importance is given for each variable in parenthesis below the variable name (based on models with ∆ AIC_C_ < 2).

| No. | Intercept | Lifespan (1) | FEC  (1) | Bill depth (0.36) | Wing length (0.38) | Bill length (0.40) | Birth year (0.20) | Tarsus length (0.08) | ∆ AIC_C_ | Weight |
| --- | --- | --- | --- | --- | --- | --- | --- | --- | --- | --- |
| 1 | - 0.51 ± 0.34 | 0.42 ± 0.15 | - 0.07 ± 0.03 | 1.25 ± 0.57 |  |  |  |  | 0 | 0.08 |
| 2 | - 0.51 ± 0.35 | 0.42 ± 0.15 | - 0.07 ± 0.03 |  |  | 0.67 ± 0.30 |  |  | 0.07 | 0.07 |
| 3 | - 0.76 ± 0.38 | 0.56 ± 0.17 | - 0.08 ± 0.03 |  | 0.18 ± 0.09 |  |  |  | 0.95 | 0.05 |
| 4 | - 0.68 ± 0.38 | 0.50 ± 0.16 | - 0.07 ± 0.03 | 0.97 ± 0.62 | 0.12 ± 0.10 |  |  |  | 1.09 | 0.04 |
| 5 | - 0.17 ± 0.44 | 0.49 ± 0.16 | - 0.08 ± 0.03 |  | 0.19 ± 0.09 |  | × |  | 1.53 | 0.04 |
| 6 | 0.08 ± 0.44 | 0.33 ± 0.15 | - 0.07 ± 0.03 |  |  | 0.63 ± 0.29 | × |  | 1.66 | 0.03 |
| 7 | - 0.58 ± 0.36 | 0.46 ± 0.16 | - 0.07 ± 0.03 |  |  | 0.59 ± 0.31 |  | 0.17 ± 0.21 | 1.98 | 0.03 |
| 8 | - 0.59 ± 0.36 | 0.46 ± 0.16 | - 0.07 ± 0.03 | 1.10 ± 0.60 |  |  |  | 0.15 ± 0.22 | 2.12 | 0.03 |
|  |  |  |  |  |  |  |  |  |  |  |
